# Dental Stem Cell Migration on Pulp Ceiling Cavities Filled with MTA, Dentin Chips, or Bio-Oss

**DOI:** 10.1155/2015/189872

**Published:** 2015-06-03

**Authors:** Stefania Lymperi, Vasiliki Taraslia, Ioannis N. Tsatsoulis, Athina Samara, Athanasios D. Velentzas, Anastasia Agrafioti, Ema Anastasiadou, Evangelos Kontakiotis

**Affiliations:** ^1^Department of Endodontics, Dental School, University of Athens, Athens, Greece; ^2^Department of Genetics and Gene Therapy, Biomedical Research Foundation of the Academy of Athens, Athens, Greece; ^3^University of Oslo, Postboks 1103, Blindern, 0317 Oslo, Norway; ^4^Department of Cell Biology and Biophysics, Faculty of Biology, University of Athens, Athens, Greece

## Abstract

MTA, Bio-Oss, and dentin chips have been successfully used in endodontics. The aim of this study was to assess the adhesion and migration of dental stem cells on human pulp ceiling cavities filled with these endodontic materials in an experimental model, which mimics the clinical conditions of regenerative endodontics. Cavities were formed, by a homemade mold, on untouched third molars, filled with endodontic materials, and observed with electron microscopy. Cells were seeded on cavities' surface and their morphology and number were analysed. The phenomenon of tropism was assessed in a migration assay. All three materials demonstrated appropriate microstructures for cell attachment. Cells grew on all reagents, but they showed a differential morphology. Moreover, variations were observed when comparing cells numbers on cavity's filling versus the surrounding dentine disc. The highest number of cells was recorded on dentin chips whereas the opposite was true for Bio-Oss. This was confirmed in the migration assay where a statistically significant lower number of cells migrated towards Bio-Oss as compared to MTA and dentin chips. This study highlights that MTA and dentin chips have a greater potential compared to Bio-Oss regarding the attraction of dental stem cells and are good candidates for bioengineered pulp regeneration.

## 1. Introduction

The treatment of pulpal necrosis of an immature tooth with an open apex has been traditionally achieved using either long term calcium hydroxide [Ca(OH)_2_] apexification procedures, or more recently the MTA apical barrier technique [1, 2]. Despite the high success rate of those treatments, further root development did not usually occur and the roots remained thin, fragile, and prone to fracture [2, 3]. Thus, new therapeutic approaches are imperative, including dental regenerative technologies.

Regenerative endodontics are referred to as biologically based procedures which aim to replace diseased, damaged, or missing structures such as dentin, root structures, and cells of the pulp-dentin complex [4]. Early experiments demonstrated the essential role of blood clot and the revascularization which provides blood supply to existing pulp tissue for subsequent completion of root development [5]. Other contributing factors are the progress in stem cell research and particularly the discovery of dental stem cells and their potential therapeutic applications on tissue engineering [6, 7].

Current studies reported the revascularization of root canals in necrotic teeth, following disinfection using antibiotics and mechanical irritation to initiate bleeding into the canal so that a blood clot would be formed at the level of cementoenamel junction. In most of these cases, mineral trioxide aggregate (MTA) was applied to seal the blood clot despite the initial difficulties to be stabilized on top of the clot [8, 9]. In fact, MTA has been used in numerous studies as a sealing pulp space barrier material in regenerative endodontics procedures [10, 11]. The main advantages of MTA are high biocompatibility, lack of cytotoxicity and carcinogenicity, antibacterial action, good sealing capacity, and radiopacity [12–14]. Furthermore, it has been shown to allow the adherence and proliferation of osteoblasts on its surface, whereas clinical studies have demonstrated that MTA could be used as a root-end filling material [15–17].

Bio-Oss is a natural, nonantigenic, porous bone mineral matrix, produced by the removal of all organic components of bovine bone. Due to its natural and chemical similarities with the human bone, it has been used in clinical practice for many years with beneficial results [18–20]. Furthermore, Bio-Oss facilitates the absorption of blood and favours the formation of vasculature due to its trabecular architecture, interconnecting macro- and micropores and its natural consistency [21, 22]. Moreover, the structure of Bio-Oss favours the migration of cells through its matrix [23].

Dentin chips, on the other hand, have been applied in a number of* in vitro* and* in vivo* studies [24–30]. This material is not commercially available and for its preparation human teeth are autoclaved and crushed into chips. The presence of dentin promotes the formation of a calcified tissue similar to ossein and accelerates healing whilst inhibiting inflammatory reaction [25, 31].

The aim of the present study is to evaluate the ability of dental stem cells extracted from Human Exfoliated Deciduous Teeth (SHEDs) to adhere, grow, and migrate on the dental pulp cavities of molars that have been filled with MTA, Bio-Oss, or dentin chips.

The advantage of our approach is that it directly compares these three reagents in a setting that simulates the clinical conditions and includes MTA, an extensively used dental reagent, as well as Bio-Oss and dentin chips that could possible serve as alternative materials for pulp space barrier sealing.

## 2. Materials and Methods

### 2.1. MOLAR Pulp Wall Processing

**Figure 1:**
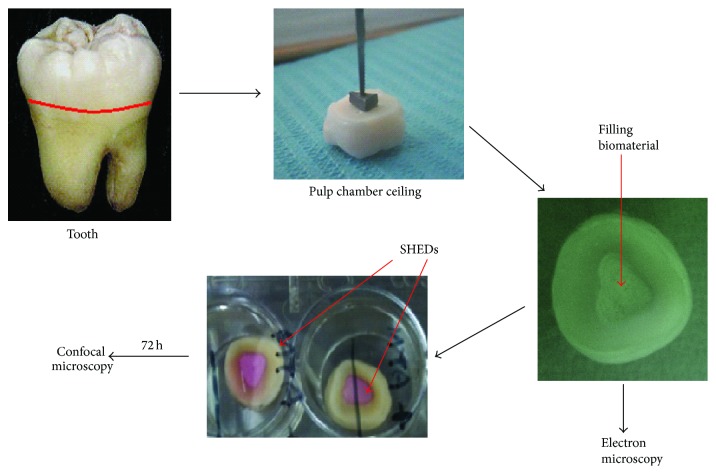
Experimental strategy. A tooth slice is prepared by removing the root of third molars and a canal is formed by using a handmade metal base. The root canal is filled with a reagent commonly used in endodontics such as MTA, Bio-Oss, or dentin chips; electron microscopy was performed to assess their surface morphology. The different materials were seeded with dental mesenchymal stem cells, cultured for 72 hours and studied by confocal microscopy; the state and the number of the cells were recorded and further analyzed.

An* in vitro* experimental model aiming to recapitulate the clinical conditions was generated in order to study the effect of three dental materials on dental mesenchymal stem cells. Twenty untouched third lower molars recently were extracted (within less than 3 months) from individuals of 20–40 years of age and each one was freshly used for the experimental assays. Each molar was cut 1 mm above the cementoenamel junction (CEJ), using a blade (Hopf, Ringleb & Co., Gmbh and Cie, Berlin, Germany) and abundance of water spray. A cavity was prepared using a handmade metal triangular base with 3 × 4 × 4 mm dimensions and 2 mm thickness. The processed samples were autoclaved and the formed cavities were filled with the different reagents. The thickness of the filling was controlled by the metal triangular construction, in a way that 1 mm of free space was formed around the cavities of the pulp wall. MTA (Dentsply Tulsa Dental Specialties, Memphis, TN) and Bio-Oss (Geistlich AG, Wolhusen, Switzerland) were prepared according to the manufacturer's instructions and five formed cavities per material were filled with the different reagents. Five samples were also filled with dentin chips and five samples were used as controls (tooth without any material) (Figure 1).

### 2.2. Dentin Chips Preparation

The crowns of ten human third molars recently extracted from individuals aged 20–40 years were autoclaved and processed as previously described [26]. Dentin chips were prepared using an autoclaved round bur US 7 RAL (Hager and Meisinger, Germany) of 2 mm diameter placed on an autoclaved glass slide without water spray and with low-speed handpiece.

### 2.3. Cell Culture

Stem cells from Human Exfoliated Deciduous Teeth (SHEDs) were provided by ProCell, Biotechnological Application SA (Athens, Greece). A 100 *µ*L drop of 10^5^ live SHEDs cell suspension was carefully placed on top of each sample and cultured for 3 days in Dulbecco's Modified Eagle's Medium (DMEM, Gibco, Glasgow, UK), supplemented with sodium pyruvate, streptomycin, penicillin, and L-glutamine and complemented with 10% heat-inactivated fetal bovine serum (FBS, Biowest) at 37°C in a 5% CO_2_ humidified incubator. Cells were used between the third and fifth passage.

### 2.4. Cell Counting

Cells cultured on the surface of the samples were fixed in 4% PFA for 20 minutes at room temperature, followed by PBS washing. The samples were then stained using Phalloidin-Rhodamine solution (Molecular Probes) at 37°C to stain the cytoskeleton and more specifically the actin filaments. 4′,6-Diamidino-2-phenylindole (DAPI) was also used for the detection of the nucleus. Samples were placed on top of a microscope slide with Vectashield (VECTOR Laboratories, Peterborough, UK) and observed under the confocal microscope Leica TCS SP5 (Leica, Wetzlar, Germany) in a 20x resolution and analysed using the Leica software, LAS AF. The whole surface of the samples was scanned under the confocal microscope and particularly the area where the different materials were in direct contact with the dentin of the pulp wall. At least five different equally sized areas were carefully observed for each sample and the cells attached on the tooth and the material were recorded and enumerated.

### 2.5. Electron Microscopy

The surface structural organization of each specimen was examined through a scanning electron microscopy (SEM) approach. Samples were attached on aluminum stubs, coated with gold/palladium in a sputter-coating apparatus (Tousimis Samsputter-2a, Rockville, Maryland, USA), for 2 min and visualized under a Philips SEM 515 scanning electron microscope.

### 2.6. Migration Assay

MTA, Bio-Oss, and dentin chips were prepared as previously described according to the manufacturer's instructions in 24-well plates and allowed to set overnight. SHEDs were cultured in serum-free DMEM for 2 hours prior to being plated to the upper chamber of the transwells. 25 × 10^3^ cells/mL were seeded within 100 mL of media into the upper compartment of a 6 mm diameter migration chamber, composed of a polycarbonate filter with 8 mm pores (Costar Transwell; Corning Inc., Corning, NY) that was prewarmed at 37°C. The cells were cultured and allowed to migrate for 6 hours at 37°C in a moist environment and 5% CO_2_. The upper chamber containing the filter was removed. The nonmigrating cells attached to the upper side of the filter were carefully removed with a cotton swab and the cells were fixed with 1% paraformaldehyde for 15 minutes at room temperature and rinsed twice with PBS. The migrating cells attached to the lower side of the filter were stained with 0.1% crystal violet (prepared in 10% ethanol) for 15 minutes at room temperature, washed with dH_2_O, and air-dried overnight. Five brightfield micrographs per sample, at 20x magnification, were randomly chosen, in order to count the migrated cells using the cell-counted plug-in of ImageJ. Results from two different experiments were expressed as mean ± SD.

### 2.7. Statistical Analysis

Statistical analysis was performed using one-way repeated analysis of variance (ANOVA). Additionally, simple univariate analysis of variance was used along with Tukey post hoc test to specify statistically significant differences, investigating all pairwise differences. For all tests, the level of statistical significance was set at 5%.

## 3. Results

**Figure 2:**
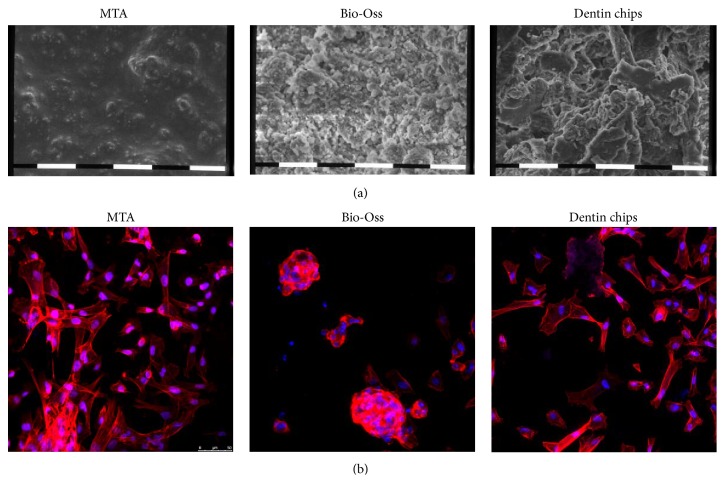
(a) The surface structural organization of each specimen was examined by scanning electron microscopy (magnification ×2200). All three reagents, MTA, Bio-Oss, and Dentin chips, offer rough surface which is adequate for cell adhesion with dentin chips showing the highest degree of coarseness followed by Bio-Oss. (b) Representative images of the cell cultures on the surface of the different dental substances by confocal microscopy. The cells were cultured for 72 hours on the provided specimens and fixed and stained with Phalloidin to depicture the cytoskeleton of the dental mesenchymal stem cells (red) and DAPI for the nucleus (blue). The morphology and the clustering of the cells vary according to the applied material.

Untouched third molars were extracted and decoronated and a cavity was made with a homemade mold as demonstrated in Figure 1. The cavities were filled with endodontic materials and, once set, cells were seeded on these surfaces and their morphology and number were analysed (Figure 1). Scanning electron microscopy (SEM) was used to study the microstructure of the MTA, dentin chips, and Bio-Oss after setting. All materials possess microstructures offering a rough surface sufficient for the viability and adhesion of dental mesenchymal stem cells (Figure 2(a)). As shown in Figure 2(a), the material with the highest porosity was the dentin chips, when compared to MTA and Bio-Oss.

Regarding cell growth and morphology, cells on MTA demonstrated a typical fibroblast-like polarized appearance with elongated cytoskeleton, whereas cells on dentin chips showed similar profile but milder spindle-shape formation (Figure 2(b)). Bio-Oss seemed to dramatically affect the cell distribution and morphology as it appeared to form compact clusters and abolished their spindle-shape appearance, becoming thus smaller and rounder with dense cytoplasm.

**Figure 3:**
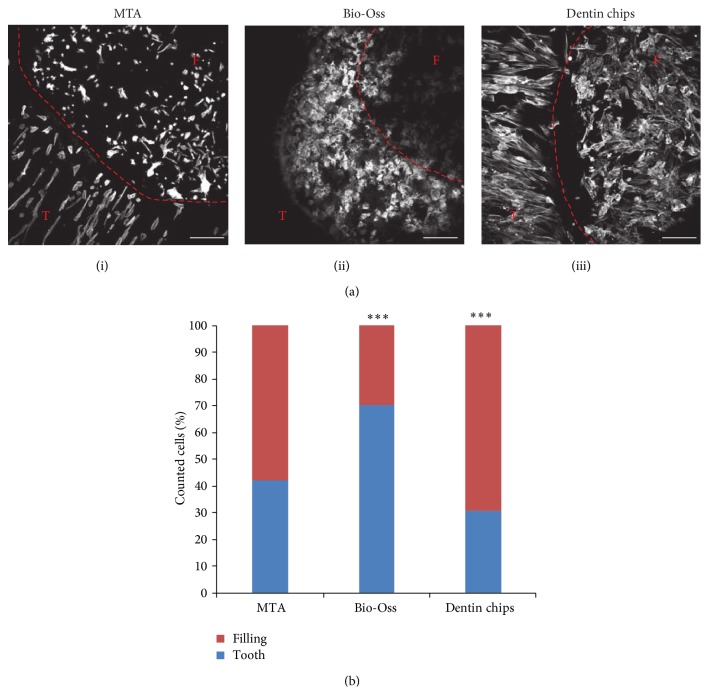
(a) Confocal images of cells showing their growing and distribution on the tooth surface and on the different root filling dental materials. (i) MTA, (ii) dentin chips, and (iii) Bio-Oss; white: Phalloidin, Bar corresponds to 250 *µ*m, T: tooth, F: filling. (b) The graph represents the percentage distribution of dental mesenchymal stem cell cultures on top of the tooth and the different filling materials. A statistically significant higher number of cells were found on dentin chips, while the opposite was true for Bio-Oss and equal distribution of the cells was observed for MTA. ^*∗∗∗*^ *p* < 0.005.

To further study dental mesenchymal stem cells in the presence of the three reagents, cells were counted in three independent experiments for each material. The analysis revealed that even though the total number of cells attached to the dental discs filled with the different materials was not statistically different among the three substrates, some materials favored the adherence and growth of dental mesenchymal stem cells on these substrates, compared to others (Figure 3(a)). More specifically, a statistically significant higher number of dental mesenchymal stem cells were found on the surface of the filling when dentin chips were analysed (One-way ANOVA, *p* = 0.009). The opposite was true for the Bio-Oss, where a statistically significant higher number of cells were found on the tooth versus the filling material (*p* = 0.005). No major differences were observed for MTA, as there was an equal distribution of cells on top of the tooth and on top of the filling (*p* = 0.252) (Figure 3(b)).

**Figure 4:**
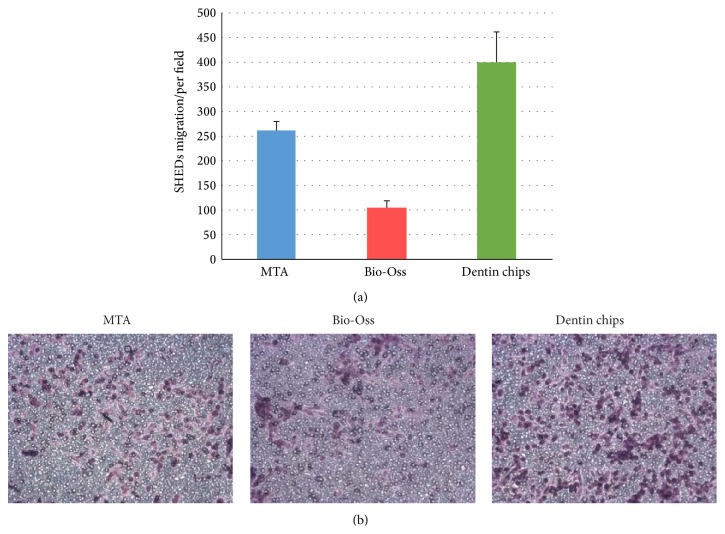
(a) Mean dental mesenchymal stem cell migration in response to the different materials. Error bars represent the standard deviation. ^*∗∗∗*^ *p* < 0.005. (b) Light microscope images at 20x magnification. The cells were fixed and stained with crystal violet to reveal their numbers and position as the cells become purple in the presence of the dye.

As the total number of cells on the dental discs is the same regardless of the different cavity filling material, it seems likely that these endodontic reagents did not demonstrate cytotoxic effects and, therefore, no major cell death or proliferation arrest was observed. This finding, in combination with the material surface SEM micrographs that demonstrated rough surfaces suitable for cell adhesion, suggests that cells might exhibit differential migratory capacity upon adherence. Indeed the above finding was further supported by the results obtained from migration assays. The three different materials were plated on migration chambers to assess whether the difference observed in the previous experiments was due to the phenomenon of tropism or taxis (Figure 4). A statistically significant higher number of cells migrated through the transwell filter when dentin chips were added in the migration chamber compared to all the other materials. Moreover, a statistically significant lower number of cells migrated through the transwell filter when Bio-Oss was added in the migration chamber compared to all the other materials. Overall, dentin chips favored the migration of cells compared to the other materials, whereas Bio-Oss inhibited the migration of dental mesenchymal stem cells as compared to MTA and dentin chips (Figure 4).

## 4. Discussion

The study of the dental stem cells responses on different substrates that could potentially be used as scaffolds for dental tissue bioengineering is fundamental for the new emerging field of regenerative endodontics. We have previously shown that ProRoot MTA is the most biocompatible material to Periodontal Ligament Fibroblasts (PDL) regarding root-end endodontic microsurgery [13]. In the current study, we have taken our research one step further by investigating the impact of the direct contact of materials that have been used both in conservative dentistry and in surgical endodontics on dental mesenchymal stem cells. Therefore, we compared MTA, a substrate traditionally used as a ceiling material in regenerative endodontics, to Bio-Oss and dentin chips, assessing whether the use of the latter could possibly serve better this role.

A successful scaffold in regenerative medicine should allow for attachment and provide the mechanical support to the cells, favouring their migration whilst enabling the circulation of biochemical factors and vital cell nutrients [4]. It is known that the effectiveness of a scaffold depends on the pore distribution, its exposed surface area, and its porosity [4]. We demonstrated by electron microscopy that all the materials analyzed in this study form a porous surface and appropriate microstructures for the attachment of cells with dentin chips exhibiting the highest porosity. This is in agreement with our finding that dentin chips provide better support to the dental mesenchymal stem cells at least when the number of cells populating this material is used as the experimental readout.

It has been previously suggested that contact of dental materials with the dentine wall may alter their properties [26]. Therefore, to overcome this limitation, we generated a model where the dental materials (MTA, Bio-Oss, and dentin chips) are in direct contact with pulp ceiling simulating the actual situation of the clinical praxis in endodontics. This* in vitro* experimental model has a number of advantages, such as the use of freshly extracted third molars, avoiding unknown conditions due to long-term storage, affecting dentin, and dental pulp cavity. Moreover, by using a handmade metal base to create the dental cavity on the dental discs, we were able to assure the homogeneity of our samples allowing our findings to be directly comparable to each other.

Using the above strategy, we demonstrated that dental mesenchymal stem cells were able to grow on the dental pulp cavity and in direct contact with all the filling materials used. This is in agreement with the previously published literature where it has been shown that human osteosarcoma cell lines, osteoblasts [16], bone marrow derived mesenchymal stem cells [32], and human tooth germ cells [33] are able to adhere and grow on MTA and similarly osteoblasts [34], mesenchymal stem cells from peripheral blood [35], or mesenchymal stem cells from bone marrow [36] can grow on Bio-Oss. Moreover, in the present study, we were able to directly compare the three materials and demonstrate for the first time that dental stem cells seemed to prefer dentin chips and MTA as opposed to Bio-Oss. In fact, as the one-way ANOVA analysis demonstrated, a statistically significant higher number of cells were found attached on dentin chips compared to the dental disc.

To further elaborate on the mechanism of this finding, we studied the cell migration towards the different materials through a transwell filter. We demonstrated that in 24 h both MTA and dentin chips attract dental mesenchymal stem cells, whereas Bio-Oss seems to inhibit their migration through the filter. Our results, regarding MTA, are in accordance with the current literature as it was previously shown that MTA promotes stem cell migration [32] and that in the presence of MTA undifferentiated dental pulp stem cells upregulated the expression of genes involved in cell migration [37]. Regarding the dentin chips, it is possible that fresh dentin chips which include organic substances [28] provide nutrients and growth factors that are important for the adherence and migration of the dental stem cells. On the contrary, Bio-Oss exhibited the poorest supporting capacity, changing their morphology to cluster formations. Given that Bio-Oss has a bovine origin [38], there might be an incompatibility issue as SHEDs are derived from human teeth. In fact, our data show that human mesenchymal stem cells perform better on human versus bovine dental discs (see Supplementary Figure in Supplementary Material available online at http://dx.doi.org/10.1155/2015/189872). Finally, MTA which is composed of inorganic materials [39] was found to preserve the morphology of dental mesenchymal stem cells and favor their adherence and growth. Further investigation needs to be performed to identify the exact molecular mechanisms that dictate the cell growth, migration, and morphology in the presence of the different dental material.

Briefly, in this comparative study and in our experimental setup, we demonstrated that dentin chips act as a superior to MTA material in regenerative endodontic procedures, promoting the adhesion and migration of dental pulp derived stem cells. However, the result of our* in vitro* studies is not easily applied in everyday clinical practice, as dentin chips are not currently commercially available. Therefore, issues of reproducible quality of the material as well as sterility and availability of the reagent should be raised. Moreover, the dentin chips preparation used throughout this study cannot be polymerized in its present form and consequently it cannot be the solid and robust sealer which once placed on top of the blood clot will form a pulp space barrier securing the dental cavity.

## 5. Conclusion

In conclusion, we demonstrated that Bio-Oss is not performing as well as MTA with respect to the adhesion and migration of dental stem cells, while dentin chips were proved to be the best performing reagent in our experimental setting. This interesting finding should be taken under serious consideration when decisions regarding root filling materials are made. Further research will serve to delineate the physiology behind these findings and their applications in regenerative endodontic procedures.

## Supplementary Material

Supplementary Figure 1: Representative images of the cell cultured on the surface of (A) human and (B) bovinedental disc by confocal microscopy. The cells were cultured for 72 hours on the provided specimens, fixed and stained with Phalloidineto depicture the cytoskeleton of the dental mesenchymal stem cells (red) and DAPIfor the nucleus (blue). The morphology and the clustering of the cells vary according to the origin of the dental disc.
