# Supplementary material for: Dental Stem Cell Migration on Pulp Ceiling Cavities Filled with MTA, Dentin Chips, or Bio-Oss
Source: Biomed Res Int. 2015 Jun 3;2015:189872. doi: 10.1155/2015/189872 (PMC4469752; doi:10.1155/2015/189872)
Supplement: Supplementary file 1 — Supplementary Figure 1: Representative images of the cell cultured on the surface of (A) human and (B) bovinedental disc by confocal microscopy. The cells were cultured for 72 hours on the provided specimens, fixed and stained with Phalloidineto depicture the cytoskeleton of the dental mesenchymal stem cells (red) and DAPIfor the nucleus (blue). The morphology and the clustering of the cells vary according to the origin of the dental disc. [file 189872.f1.pdf]

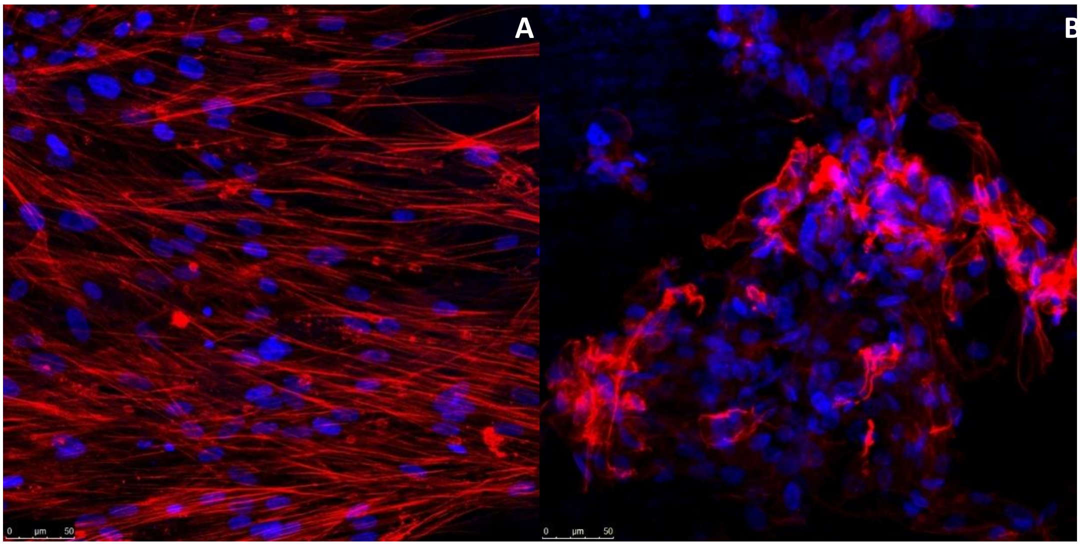

Supplementary figure 1: Representative images of the cell cultured on the surface of **(A)** human and **(B)** bovinedental disc by confocal microscopy. The cells were cultured for 72 hours on the provided specimens, fixed and stained with Phalloidineto depicture the cytoskeleton of the dental mesenchymal stem cells (red) and DAPIfor the nucleus (blue). The morphology and the clustering of the cells vary according to the origin of the dental disc.
